# Radiological analysis of the sagittal profile of the Colombian population according to the theoretical Roussouly classification

**DOI:** 10.1186/s12891-026-09700-5

**Published:** 2026-04-29

**Authors:** Juan Esteban Muñoz Montoya, Ajoy Prasad Shetty, Rajasekaran Shanmuganathan

**Affiliations:** 1 Nueva Granada Military University, Bogotá, Colombia; 2 Ganga Hospital, 313 Mettupalayam Road, Coimbatore, Tamil Nadu 641043 India

**Keywords:** Hispanic, Sagittal profile, Spinal curvatures, Roussouly classification

## Abstract

**Background:**

The theoretical Roussouly classification describes sagittal spinal profiles based on pelvic morphology. As most reference data originate from Caucasian populations, its applicability to Hispanic populations remains limited. This study aimed to describe sagittal spinal alignment in an asymptomatic Colombian population using the theoretical Roussouly classification.

**Methods:**

A retrospective cross-sectional study analyzed whole-spine standing radiographs (2020–2024) from asymptomatic adults aged 18–50 years at Clínica Tolima. Spinopelvic parameters were measured using Surgimap, and sagittal profiles were classified according to the theoretical Roussouly classification.

**Results:**

A total of 792 participants (57.58% female) were included. All sagittal profile types were identified. Type 3 was the most prevalent (36.74%), followed by Type 4 (28.16%); Type 3 Anteverted Pelvis (AP) accounted for 11.11%. Strong correlations were observed between Pelvic Incidence (PI) and Sacral Slope (SS) (*r* = 0.82), PI and Lumbar Lordosis (LL) (*r* = 0.88), and SS and LL (*r* = 0.79) (all *p* < 0.001). Sex was associated with higher SS and LL values, while age showed a limited association with SS only.

**Conclusions:**

Sagittal profile distribution and spinopelvic relationships in this Colombian population were comparable to those reported in other ethnic groups.

## Introduction

Sagittal spinal alignment is central to understanding spinopelvic biomechanics in both asymptomatic individuals and patients with spinal pathology, reflecting the interaction between the spine and pelvis to maintain balance [1, 2]. Pelvic parameters described by Duval-Beaupère et al., including PI, Pelvic Tilt (PT), and SS, are fundamental to defining sagittal pelvic morphology in healthy individuals [3].

However, variability in spinal sagittal alignment means that Pierre Roussouly et al. classify four spinal sagittal profiles (spinal shapes) in normal individuals based on SS [4]. Roussouly et al. identified 160 normal, asymptomatic Caucasian patients, of whom 34 had an SS < 35° associated with low PI and 48 had an SS > 45° associated with high PI. He also found a strong correlation between SS and PI (*r* = 0.080) and a moderate correlation between PT and PI (*r* = 0.65). This classification was modified and clarified by Laouissat in 2017, which included a fifth type of sagittal profile (Type 3 AP), used the PI to classify sagittal profiles, and changed toward the Theoretical Roussouly Classification [5].

Previous studies have applied the classical Roussouly classification to describe sagittal spinal profiles in Caucasian [4, 5], Asian populations, including Chinese [6] and Korean cohorts [7], as well as in multiethnic analyses [8]. In Latin America, data remain limited. Yamazato et al. [9] evaluated the reliability of the Roussouly classification in a Brazilian population, while Guiroy et al. [10] described the distribution of sagittal profile types in a small asymptomatic Argentine cohort, without detailed analysis of spinopelvic relationships.

Restoration of sagittal alignment to an individual’s native profile has been associated with reduced complications and improved long-term outcomes in adult spinal deformity surgery [11, 12]. Accordingly, this study aimed to classify sagittal spinal profiles in an asymptomatic Colombian population using the theoretical Roussouly classification, providing reference data for normal sagittal alignment.

## Methods

### Study design and population

**Fig. 1:**
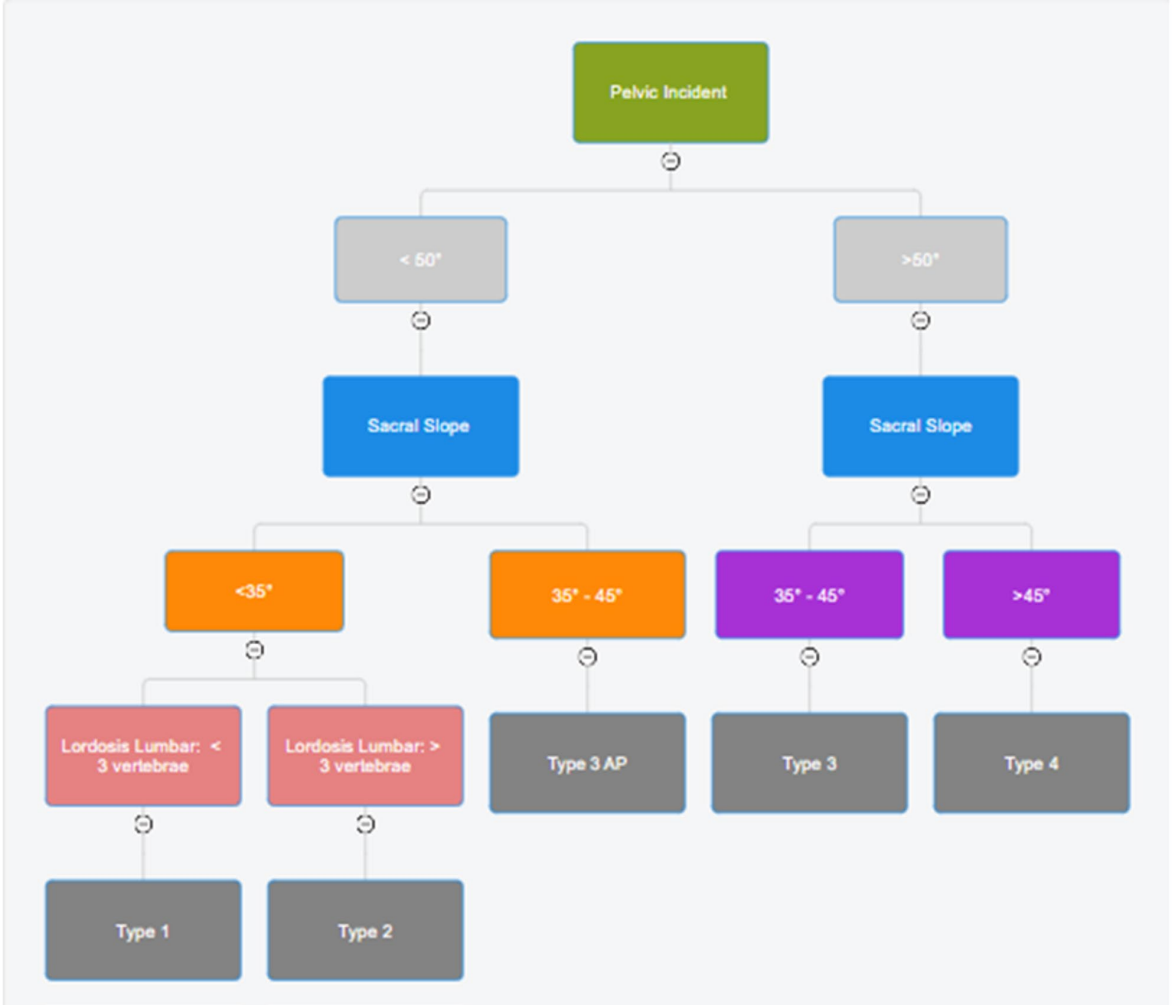
Algorithm of the theoretical Roussouly classification to classify the study participants

This retrospective cross-sectional study evaluated sagittal spinal alignment in an asymptomatic Colombian (Hispanic) population using the theoretical Roussouly classification (Fig. 1). The analysis was based on previously acquired whole-spine standing radiographs obtained between January 2020 and December 2024 at Clínica Tolima. Radiographic and demographic data were retrieved from institutional databases, and no participant follow-up was performed.

Given the analytical comparisons across the theoretical Roussouly types, sex, and age groups, the sample size was estimated using a cohort-based formula that assumes an infinite population, yielding a minimum required sample of 280 participants. The final sample was expanded to improve statistical precision.

Participants were recruited from the outpatient department and included individuals undergoing routine occupational or institutional medical evaluations. Inclusion criteria were asymptomatic adults aged 18–50 years. Exclusion criteria comprised spinal deformity, prior spinal surgery, recent trauma or infection, and systemic musculoskeletal conditions, including Parkinson’s disease, osteoporosis, autoimmune disorders, or connective tissue diseases.

This Study complies with the STROBE statement checklist for retrospective and cross-sectional studies.

### Radiographic evaluation and classification of types

Whole-spine standing radiographs were obtained with participants in a relaxed upright position, barefoot, knees extended, and gaze directed horizontally. Arms were flexed at approximately 30° with hands resting on the clavicles to avoid superimposition. Images included the spine from C1 to the femoral heads using a 14″ × 36″ digital cassette and a 180-cm source–image distance (Carestream or Siemens systems). The Sagittal alignment parameters—Pelvic Incidence (PI), Sacral Slope (SS), Pelvic Tilt (PT), and Lumbar Lordosis (LL)—were measured using Surgimap. Radiographs were classified according to the theoretical Roussouly classification into five types: Types 1–4 and Type 3 Anteverted Pelvis (3AP).

Two independent observers performed all measurements and classifications. A third evaluator resolved discrepancies. Interobserver reliability was excellent, with a weighted kappa (k) of 0.99 for the theoretical Roussouly classification and intraclass correlation coefficients (ICC) ranging from 0.89 to 0.97 for sagittal parameters.

### Statistical analysis

Descriptive statistics were calculated for all variables using Surgimap to ensure standardized radiographic measurements. Quantitative variables are presented as means and standard deviations, and qualitative variables as frequencies and proportions. The data distribution was assessed using the Shapiro–Wilk test. Given the large sample size (*n* > 30 per theoretical Roussouly type), normality assumptions were additionally supported by the Central Limit Theorem.

Comparisons of the theoretical Roussouly classification across sex and age groups were performed using the Chi-square test. Differences in spinopelvic parameters (PI, SS, PT, and LL) by sex, age group, and theoretical Roussouly type were analyzed using one-way ANOVA, with 95% confidence intervals reported.

Associations between spinopelvic parameters were assessed using Pearson or Spearman correlation coefficients, as appropriate. Finally, multivariable linear regression analyses were conducted in R (version 4.0.5) to evaluate the effects of age and sex on spinopelvic parameters.

### Ethical considerations

This study used data from a larger research project approved by the Institutional Ethics Committee of Clínica Tolima (approval No. 108, 19/09/2024) and the Hospital Bioethics Committee under the Kawak Integrated System.

## Results

### Demographic characteristics and theoretical Roussouly distribution

A total of 792 asymptomatic Colombian adults were included, with a mean age of 34.2 years (range 18–50); 57.58% were female (*n* = 456). All theoretical Roussouly sagittal profiles were identified. The most prevalent pattern was Type 3 (36.74%), followed by Type 4 (28.16%). Type 3 Anteverted Pelvis (3AP) accounted for 11.11% of the sample (Table 1).

**Table 1:** Demographic characteristics of the population

Variables	*N* = 792
Age in years, mean (SD)	18–50 years 34.16 (9.64)

Sex	Mean	SD	*p*-value
Female	34,5	9,7	0,278
Male	33,7	9,6	
Total	34,2	9,7	

Population		*n*	%	Chi-sq proportions *p*-value
Sex	Female	456	57,58%	0,000
	Male	336	42,42%	
Age groups	18–27 years	241	30,43%	0,043
	28–39 years	263	33,21%	
	40–50 years	288	36,36%	
Theoretical Roussouly Classification	1	135	17,05%	0,000
	2	55	6,94%	
	3	291	36,74%	
	4	223	28,16%	
	3 AP	88	11,11%	
Total		792	100,00%	

*SD*  Standard Deviation, *AP*  Anteverse Pelvis

### Theoretical Roussouly classification according to age and sex

Across age groups, Type 3 remained the predominant sagittal profile (36.9% in 18–27 years, 41.1% in 28–39 years, and 32.6% in 40–50 years), with no significant differences in distribution (Table 2). When stratified by sex, Type 3 was also the most frequent pattern in both males and females. However, a statistically significant difference in overall distribution was observed, indicating a distinct sex-related sagittal profile pattern (Table 3).

**Table 2:** Distribution of the theoretical Roussouly classification according to age groups

**Theoretical ** **Roussouly Classification**	**18 – 27 ** **years**		**28 – 39 ** **years**		**40 – 50 ** **years**		**Total**		**Chi-sq Test**
	n	%	n	%	n	%	n	%	
1	45	18,7%	37	14,1%	53	18,4%	135	17,0%	0,262
2	16	6,6%	18	6,8%	21	7,3%	55	6,9%	
3	89	36,9%	108	41,1%	94	32,6%	291	36,7%	
4	63	26,1%	66	25,1%	94	32,6%	223	28,2%	
3 AP	28	11,6%	34	12,9%	26	9,0%	88	11,1%	
Total	241	100,0%	263	100,0%	288	100,0%	792	100,0%

**Table 3:** Distribution of the theoretical Roussouly classification according to sex

**Theoretical ** **Roussouly Classification **	**Female**		**Male**		**Total **		**Chi-sq Test**
	n	%	n	%	n	%	
1	66	14,5%	69	20,5%	135	17,0%	0,023
2	28	6,1%	27	8,0%	55	6,9%	
3	162	35,5%	129	38,4%	291	36,7%	
4	143	31,4%	80	23,8%	223	28,2%	
3 AP	57	12,5%	31	9,2%	88	11,1%	
Total	**456**	**100%**	**336**	**100%**	**792**	**100%**	

*Abbreviations*: *AP* Anteverse Pelvis

### Spinopelvic parameters by theoretical Roussouly type

Mean values of pelvic incidence (PI), sacral slope (SS), pelvic tilt (PT), and lumbar lordosis (LL) differed significantly across theoretical Roussouly types (Table 4). A progressive increase in PI, SS, and LL was observed from Type 1 to Type 4, consistent with the morphologic continuum described in the original Roussouly classification. Conversely, PT demonstrated an inverse relationship with SS. The Type 3AP subgroup showed intermediate PI and SS values, lower PT consistent with anterior pelvic rotation, and LL values between those observed in Types 3 and 4.

**Table 4:** Distribution of spinopelvic parameters by theoretical Roussouly classification

Parameters	Theoretical Roussouly Classification	*n*	Mean (°)	Min (°)	Max (°)	*p* -value
PI	1	135	39,6	38,3	40,8	0,000
	2	55	43,1	41,2	45,1	
	3	291	56,9	56,0	57,7	
	4	223	66,8	65,9	67,8	
	3 AP	88	43,4	41,9	45,0	
SS	1	135	27,3	26,4	28,3	0,000
	2	55	31,4	29,9	32,8	
	3	291	38,7	38,0	39,3	
	4	223	51,6	50,9	52,4	
	3 AP	88	39,5	38,4	40,7	
PT	1	135	12,2	11,3	13,2	0,000
	2	55	11,2	9,7	12,8	
	3	291	17,9	17,3	18,6	
	4	223	15,7	14,9	16,4	
	3 AP	88	3,7	2,5	4,9	
LL	1	135	45,9	44,3	47,4	0,000
	2	55	47,5	45,1	50,0	
	3	291	64,8	63,7	65,9	
	4	223	75,7	74,4	76,9	
	3 AP	88	56,2	54,2	58,1	

*Abbreviations*: *PI *Pelvic Incidence, *SS *Sacral Slope, *PT *Pelvic Tilt, *LL *Lumbar Lordosis, *Min *Minimus, *Max *Maximus, *AP *Anteverse Pelvis

### Overall spinopelvic alignment and stratified analyses

In the overall cohort, mean spinopelvic values were PI 54.3°, SS 40.0°, PT 14.3°, and LL 62.5° (Table 5). When analyzed by sex, lumbar lordosis was significantly greater in females, whereas no clinically relevant differences were observed for PI, SS, or PT (Table 6). Across age groups, spinopelvic parameters remained largely stable, with only minimal variation in SS, suggesting limited age-related influence on sagittal alignment within this asymptomatic adult population (Table 7).

**Table 5:** Descriptive Statistics of Lumbar Lordosis and Pelvic Parameters in the Overall Sample

Parameters	*n*	Mean (°)	CI 95% (°)	SD (°)	Max (°)	Min (°)	SW *p*-value
PI	792	54,3	54,29 +/-0,45	12,6	114	17	0,000
SS	792	40,0	39,98+/-0,36	10,1	90	10	0,000
PT	792	14,3	14,28+/-0,26	7,2	35	-15	0,000
LL	792	62,5	62,47+/-0,5	14,2	116	24	0,003

**Table 6:** Comparison of Lumbar Lordosis and Pelvic Parameters between Males and Females

	Sex	*n*	Mean (°)	SD (°)	Max (°)	Min (°)	SW *p*-value	ANOVA *P* value
PI	Female	456	54,69	0,58	97	23	0,038	0,303
	Male	336	53,75	0,71	114	17	0,000	
SS	Female	456	40,64	0,46	78	14	0,011	0,032
	Male	336	39,09	0,56	90	10	0,000	
PT	Female	456	14,12	0,35	40	-15	0,001	0,460
	Male	336	14,50	0,38	35	-5	0,029	
LL	Female	456	63,64	0,63	113	27	0,059	0,007
	Male	336	60,89	0,82	116	24	0,069

**Table 7:** Comparison of Lumbar Lordosis and Pelvic Parameters between the age groups

Parameters	Years	n	Mean (°)	SD (°)	Max (°)	Min (°)	SW p-value	ANOVA P value
PI	18 – 27	241	53,35	0,81	114	25	0,000	0,382
	28 - 39	263	54,67	0,79	97	17	0,003	
	40 - 50	288	54,73	0,74	90	26	0,100	
SS	18 – 27	241	38,67	0,64	90	12	0,000	0,051
	28 - 39	263	40,70	0,63	78	10	0,002	
	40 - 50	288	40,41	0,59	70	15	0,224	
PT	18 – 27	241	14,79	0,45	40	-10	0,024	0,418
	28 - 39	263	14,07	0,46	35	-15	0,024	
	40 - 50	288	14,04	0,43	35	-6	0,040	
LL	18 – 27	241	61,35	0,89	110	24	0,195	0,320
	28 - 39	263	63,18	0,92	116	25	0,109	
	40 - 50	288	62,77	0,82	100	27	0,104	

*Abbreviations*: *PI *Pelvic Incidence, *SS *Sacral Slope, *PT *Pelvic Incidence, *LL *Lumbar Lordosis, *n *Sample, *SD *Standard Deviation, *Max *Maximus, *Min *Minimums

### Correlations among spinopelvic parameters

The correlation in Table 8 shows the relationships among lumbar lordosis (LL) and pelvic parameters (PI, SS, and PT). Pelvic Incidence (PI) demonstrated a robust positive correlation with Sacral Slope (SS) (*r* = 0.82, *p* = 0.000) and a strong positive correlation with Pelvic Tilt (PT) (*r* = 0.60, *p* = 0.000). Lumbar Lordosis (LL) also showed a robust positive correlation with PI (*r* = 0.88, *p* = 0.000) and SS (*r* = 0.79, *p* = 0.000).

**Table 8:** Pearson Correlation Matrix between Lumbar Lordosis (LL) and Pelvic Parameters (PI, SS, PT)

Parameters	PI		SS		PT		LL	
	*r*	*p* -value	*r*	*p* -value	*r*	*p* -value	*r*	*p* -value
SS	0,82	0,000	1					
PT	0,60	0,000	0,07	0,051	1			
LL	0,88	0,000	0,79	0,000	0,42	0,000	1	

*Abbreviations*: *PI* Pelvic Incidence, *SS* Sacral Slope, *PT* Pelvic Tilt, *LL* Lumbar Lordosis

### Multivariable regression analysis

Multivariable linear regression showed that sex was independently associated with SS and LL, with higher values observed in females (Table 9). Age was associated with SS but not with LL. However, the explanatory power of the models was low (R² ≈ 0.01), indicating that although age and sex contribute statistically to variation in sagittal parameters, most of the variability is likely explained by other anatomical or biomechanical factors.

**Table 9:** Multivariable Linear Regression Model for Dependent and Independent Variables

Overall Fit		Sex	Age	Sacral Slope (SS)				
Multiple R	0,1119		coeff	*p*-value	vif	*p*-value	Breusch-Pagan	
R Square	0,0125	Intercept	34,71	0,000		0,007	*p*-value	0,813
Adjusted R Square	0,0100	Sex	1,49	0,039	1,00			
Standard Error	10,0291	Age	0,09	0,021	1,00			
Observations	792							
Multiple R	0,11	Sex	Age	Lumbar Lordosis (LL)				
R Square	0,01		coeff	*p*-value	vif	*p*-value	Breusch-Pagan	
Adjusted R Square	0,01	Intercept	55,80	0,000		0,010	*p*-value	0,065
Standard Error	14,12	Sex	2,69	0,008	1,00			
Observations	792	Age	0,07	0,173	1,00			

The multivariable regression analysis revealed significant associations between age and sex with Sacral Slope (SS), and between Lumbar Lordosis (LL) and sex, predominantly among female participants

## Discussion

Numerous studies have investigated humans’ sagittal balance and normal sagittal parameters [3, 13]. Due to the significant variability among humans in spinopelvic parameters and sagittal profiles, Roussouly et al. proposed a classification system in 2005 for the different types of sagittal shapes [4].

Roussouly et al. reported Type 3 as the most frequent sagittal profile and Type 2 as the least frequent in 160 asymptomatic Caucasian volunteers [4]. Similar distributions have been described in Asian populations, including Korean [6] and Chinese cohorts [7], where Type 3 also predominated. In Latin America, Guiroy et al. [10] reported comparable findings in an Argentine cohort of 100 asymptomatic individuals. In the present Colombian population, Type 3 was likewise the most frequent profile (36.7%), whereas Type 2 was the least common (6.9%). Overall, these findings suggest a consistent distribution of sagittal profiles across Caucasian, Asian, and Hispanic populations. Table 10 summarizes the comparative studies.

**Table 10:** Historical analysis of the standardization of Roussouly’s classification and modification of Laouissat over time and the population groups studied

Spinal Shape or Sagittal Profile	Roussouly: 2005 (4)	Laouissat: 2017 (5)	Panpan Hu et al. 2016 (6)	Yongjae Cho 2017 (7)	Guiroy te al 2018 (10)	MEANS Study: Roussouly Current vs. Theoretical 2024 (8)	Muñoz Montoya et al. 2024
Population	*n* = 160 Caucasians	*n* = 296 Caucasians	*n* = 272 Chinese	*n* = 252 Koreans	*n* = 100 Argentines (Hispanic)	*n* = 467 France: 98/467 Japan: 119/467 Singapore: 79/467 Tunisia: 80/467 United States: 91/467	*n* = 792 Colombians (Hispanics)
Type 1	34 -SS: <35 (21°- 35°) -PI: 41 (34–54) - LL: 52° (41°- 64°)	12% from the population - SS: 29° ± 4° - PI: 39° ± 5° - LL: 51° ± 6° - PT: 10° ± 5°	63 (23.2%) -SS: 29.8° ± 4.2° -PI: 40.3° ± 7.3° -LL: 46° ± 6.3° -PT: 10.5° ± 6.9°	58 (23%) -SS: 22.8° ± 3.1° -PI: 38.3° ± 2.6° -LL: 47.8° ± 9.3° -PT: 17.6° ± 3.9°	18 participants -	35 (7.5%) SS: 27.2° ± 5.5° PI: 40.8° ± 9.2° LL: 43.7° ± 9.8° PT: 13.6° ± 8.9° (Current)	144 (18.2%) -SS: 25° (24° − 26.3°) -PI: 39.7° (38.5° − 41°) -LL: 41.1° (38.6°- 44°) -PT: 14.5° (13.4° − 15°)
Type 2	18 -SS: <35 (28°- 35°) -PI: 44 (38–57) -LL: 52 (44° − 58°)	22% from the population - SS: 30° ± 4° - PI: 41° ± 6° - LL: 48° ± 5° - PT: 10° ± 5°	38 (14%) SS: 31.4° ± 2.8° PI: 43.1° ± 8.6° LL: 38.8° ± 5.9° PT: 11.7° ± 6.7°	33(13.1%) -SS: 30.4° ± 2.4° -PI: 46.1° ± 8.6° -LL: 37.8° ± 5.5° -PT: 18.6° ± 6.7°	20 participants -	100 (21.4%) SS: 30.6° ± 3.7° PI: 43.6° ± 7.6° LL: 46.8° ± 8.5° PT: 13° ± 7.2° (Current)	52 (6.6%) SS: 30.3° (28.6° − 31.9°) PI: 43° (41.7° − 44.3°) LL: 24.3° (14.1°- 34.4°) PT: 12.1° (10.4° − 13.8°)
Type 3	60 - SS: 35°- 45° (39°) - PI: 51 (36–65) - LL: 61 (43° − 76°)	30% from the population - SS: 39° ± 3° - PI: 53° ± 7° - LL: 58° ± 10° - PT: 13° ± 7°	130 (47.8%) SS: 38.9° ± 2.8° PI: 47.6° ± 6.4° LL: 53.5° ± 6.7° PT: 8.6° ± 6.5°	125(49.6%) -SS: 38.9° ± 2.8° -PI: 47.6° ± 6.4° -LL: 53.5° ± 6.7° -PT: 13.8° ± 2.1°	49 participants -	222 (47.5%) SS: 40.1° ± 2.7° PI: 52.4° ± 7.6° LL: 58.9° ± 7.3° PT: 12.3° ± 7.1° (Current)	310 (39.1%) SS: 36.4° (35.8° − 37°) PI: 57.4° (56.6° − 58.1°) LL: 51.7° (49.7°- 53.7°) PT: 21° (20.2° − 21.8°)
Type 4	48 - SS: >45° (45°- 66°) - PI: 63 (43–83) - LL: 71° (61° − 82°)	20% from the population - SS: 49° ± 4° - PI: 62° ± 8° - LL: 69° ± 6° - PT: 12° ± 7°	41 (15.1%) SS: 51.3° ± 4.3° PI: 59.9° ± 9.8° LL: 65.1° ± 5.8° PT: 8.5° ± 9.6°	36(14.3%) -SS: 53.7° ± 5.1° -PI: 60.4° ± 6.8° -LL: 67.5° ± 5.6° -PT: 17.08° ± 7.7°	13 participants -	110 (23.6%) SS: 50.3° ± 4° PI: 62.4° ± 8.6° LL: 68.5° ± 7.1° PT: 12.2° ± 7.2° (Current)	204 (25.8%) SS: 49.9° (48.9° − 50.9°) PI: 66.7° (65.2° − 68.1°) LL: 62.1° (59.3°- 65°) PT: 17.7° (16.3° − 19°)
Type 3AP	-	16% of the population - SS: 44° ± 6° - PI: 48° ± 6° - LL: 64° ± 7° - PT: 4° ± 3°	-	-	-	52(11.1%) PI: 43.7° ± 5° (Current)	82 (10.3%) SS: 38.7° (37.4° − 40°) PI: 43.9° (42.6° − 45.1°) LL: 51.9° (47.3°- 56.5°) PT: 5.5° (3.7° − 7.2°)

Laouissat et al. reported the Type 3AP variant in 16% of an asymptomatic Caucasian population, characterized by intermediate PI and high SS values [5]. In the present Colombian cohort, Type 3AP was identified in 11.11% of participants, was more frequent in women, and showed comparable spinopelvic characteristics. Overall, these findings suggest similar morphologic behavior of the 3AP subtype across Caucasian and Hispanic populations. Additionally, multivariable analysis demonstrated that sex was associated with both SS and LL, whereas age was only weakly related to SS.

The PI has been identified as the key parameter regulating sagittal balance [3]. Consistent with prior studies by Roussouly and Legaye et al. [3, 5], the present Colombian cohort showed similar relationships between PI, SS, and PT, supporting comparable spinopelvic behavior across Caucasian and Hispanic populations.

The Roussouly classification is clinically relevant because it helps identify biomechanical stress patterns associated with specific degenerative pathways [14]. Previous studies have shown that Types 1 and 2 are more frequently associated with disc degeneration and herniation. In contrast, Types 3 and 4 are linked to facet degeneration and spinal stenosis, with Type 3 generally considered the most balanced and least symptomatic profile [4, 15]. Furthermore, it has been validated as a predictor of surgical outcomes and mechanical complications, including adjacent segment disease and revision surgery in short fusions and adult spinal deformity. Failure to restore the patient’s ideal sagittal profile has been associated with a markedly increased risk of mechanical complications [15–17].

The MEANS study (2024) compared theoretical and current Roussouly classifications across multiple populations and found that approximately one-third of asymptomatic individuals differed between the two systems, particularly in the presence of greater PI–LL mismatch. These findings suggest that when applied to deformity surgery, the Roussouly classification should be interpreted in the context of the patient’s current sagittal alignment and clinical status [8].

The study’s limitations were due to its cross-sectional design, which observed only a specific population and did not examine any population with pathology. Another limitation was the failure to exhaustively search for degenerative pathology with spinal Magnetic Resonance Imaging (MRI), as it included only patients with radiographs.

## Conclusion

Conducting a historical analysis of the theoretical Roussouly classification of sagittal profiles across populations, this study observed similar behavior in the asymptomatic Caucasian and Hispanic populations. Furthermore, it showed a similar correlation between Pelvic parameters and Lumbar Lordosis compared with that reported in Roussouly’s classical study.

### Ethical considerations

The study was conducted in accordance with the Declaration of Helsinki. It was approved by the Institutional Ethics Committee (IEC) of Clinica Tolima, Ibague, Colombia (No. 108 − 19/09/2024), and by the Hospital Bioethics Committee of our institution under the Kawak integrated system. This study is not a clinical trial; it was approved, and informed consent was waived due to its retrospective nature.

## Data Availability

The datasets used and/or analyzed during the current study are available from the corresponding author upon reasonable request to Ajoy Prasad Shetty: ajoyshetty@gmail.com o Juan Esteban Muñoz Montoya: juanesteban1285@gmail.com.

## Abbreviations

AP: Anteverse Pelvis
